# Electronic Circuit Simulations as a Tool to Understand Distorted Signals in Single-Entity Electrochemistry

**DOI:** 10.1021/acs.jpclett.2c02720

**Published:** 2022-10-21

**Authors:** Kannasoot Kanokkanchana, Kristina Tschulik

**Affiliations:** † Chair of Analytical Chemistry II, Faculty of Chemistry and Biochemistry, ZEMOS 1.45, Ruhr University Bochum, Universitätsstraße 150, D-44780Bochum, Germany; ‡ Max-Planck-Institut für Eisenforschung GmbH, Max-Planck-Straße 1, Düsseldorf40237, Germany

## Abstract

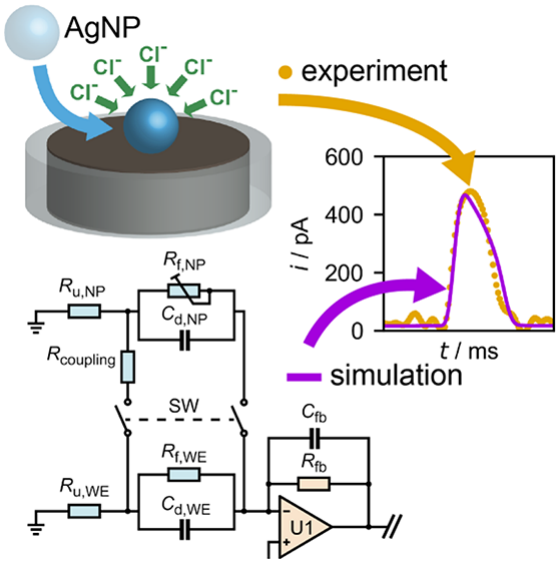

Electrochemical analysis relies on precise measurement of electrical signals, yet the distortions caused by potentiostat circuitry and filtering are rarely addressed. Elucidation of these effects is essential for gaining insights behind sensitive low-current and short-duration electrochemical signals, e.g., in single-entity electrochemistry. We present a simulation approach utilizing the Electrical Simulation Program with Integrated Circuit Emphasis (SPICE), which is extensively used in electronic circuit simulations. As a proof-of-concept, we develop a universal electrical circuit model for single nanoparticle impact experiments, incorporating potentiostat and electronic filter circuitry. Considering these alterations, the experimentally observed transients of silver nanoparticle oxidation were consistently shorter and differently shaped than those predicted by established models. This reveals the existence of additional processes, e.g., migration, partial or asymmetric oxidation. These results highlight the SPICE approach’s ability to provide valuable insights into processes occurring during single-entity electrochemistry, which can be applied to various electrochemical experiments, where signal distortions are inevitable.

The emerging field of “single-entity electrochemistry” (SEE) refers to measurement and interpretation of the electrical transient signals generated when a single entity, e.g., a nanoparticle, a single molecule, a droplet, a micelle, or a living cell, undergoes electrochemical processes at suitable interfaces, including micro- and nano-electrodes or -pipets, nanopores, etc.^1−5^ The technique’s capability to probe a signal from single entities makes it powerful for delivering both individual and statistical information on these entities. This makes it beneficial for a broad range of investigations, including nanoparticle characterizations and dynamic transformations,^6−9^ agglomeration study,^10^ ion diffusion and solvation studies,^11,12^ detection and identification of single bacteria or viruses,^13−15^ catalytic activity investigation of single enzymes,^16^ detection of conformation changes of a single DNA,^17^ single-molecule detection,^18^ battery material characterization,^19^ and investigation of the catalytic activity of individual nanoparticles.^20−23^

Despite its promising future, measuring and interpreting the transient electrochemical signals from SEE currently presents a technological challenge. In contrast to conventional electrochemistry, SEE deals with low-current and short-duration electrochemical signals that are readily affected by the instrument’s circuitry and filtering required for the practical experiments.^24,25^ For instance, Kätelhön et al. proposed mechanistic models for a single spherical nanoparticle electrooxidation at a microelectrode during nano impact experiments. In this subset of SEE experiments, a signal is detected when a nanoparticle in a suspension randomly collides with an electrode (Figure 1A).^4,26^ The authors also demonstrated that the modeled peak shapes and durations cannot be experimentally verified, since the severe distortions of the experimental impact signals obscure any interpretable spike characteristics.^26^ Since instrumentation’s internal circuitry contributes in distortions, the experimental spikes cannot be easily simulated solely using conventional electrochemical simulation tools. Due to the lack of an effective method for directly analyzing distorted nano impact data, the majority of nano impact experiments to date have focused on analyzing charge (via the integral area of the *I*–*t* spike) or limiting current, the former of which is known to be preserved during the measurement (Figure 1B).^24,26^

**Figure 1:**
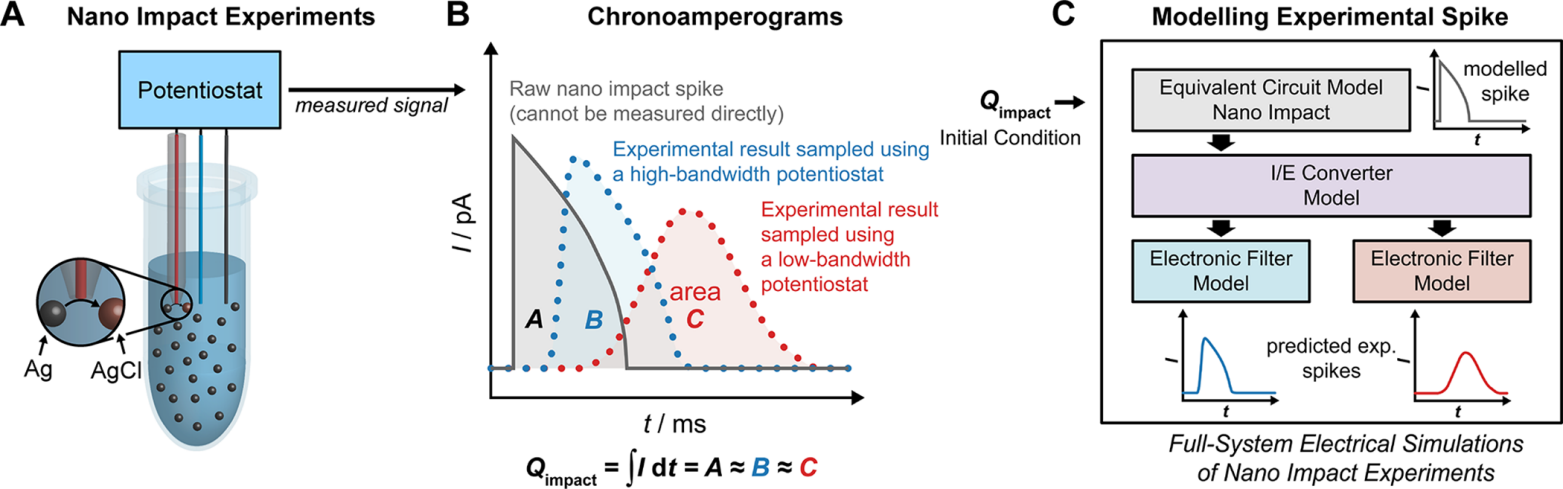
(A) An illustration of a nano impact electrochemical experiment in which a current spike is generated upon a collision of each nanoparticle; (B) instrumentation-induced signal distortions make data interpretation complicated even when the charge *Q*_impact_ is generally well conserved; (C) by constructing a full-system electrical model for the nano impact experiment that incorporates instrumentation effects, direct simulations of the distorted experimental signals are possible using *Q*_impact_ as an initial condition, providing further insight into the experimental data.

To understand and gain useful insights behind the transitory signals from SEE experiments, we require a more powerful approach for interpreting experimentally distorted data. The goal of this work is to showcase such an approach by applying electrical simulations to predict distortions caused by instrumentation and filtering during SEE measurements. First, we focus on the development of a generic simulation model for the nano impact experiments that is capable of predicting experimental spike distortions. This could be achieved by full-system simulations of an electrical equivalent circuit model of nano impact experiments in conjunction with electrical circuit models of a current-to-voltage (I/E) converter and an electronic low-pass filter (LPF) utilized in potentiostats. If successful, we could directly compare simulation results to distorted experimental data to gain insights into these experimental nano impact signals (Figure 1C).

The generalized simulation model for the nano impact experiments is shown in Figure 2. It comprises three circuit blocks: an electrical equivalent circuit model of the nano impact electrochemical cell (Figure 2A), an electrical model for the I/E converter (Figure 2B), and an electrical model of the LPF (Figure 2C). These three basic blocks may be configured with a variety of component values to match the dynamic response of the experimental setup, which can be experimentally analyzed using the approach described in our previous work.^24^

**Figure 2:**
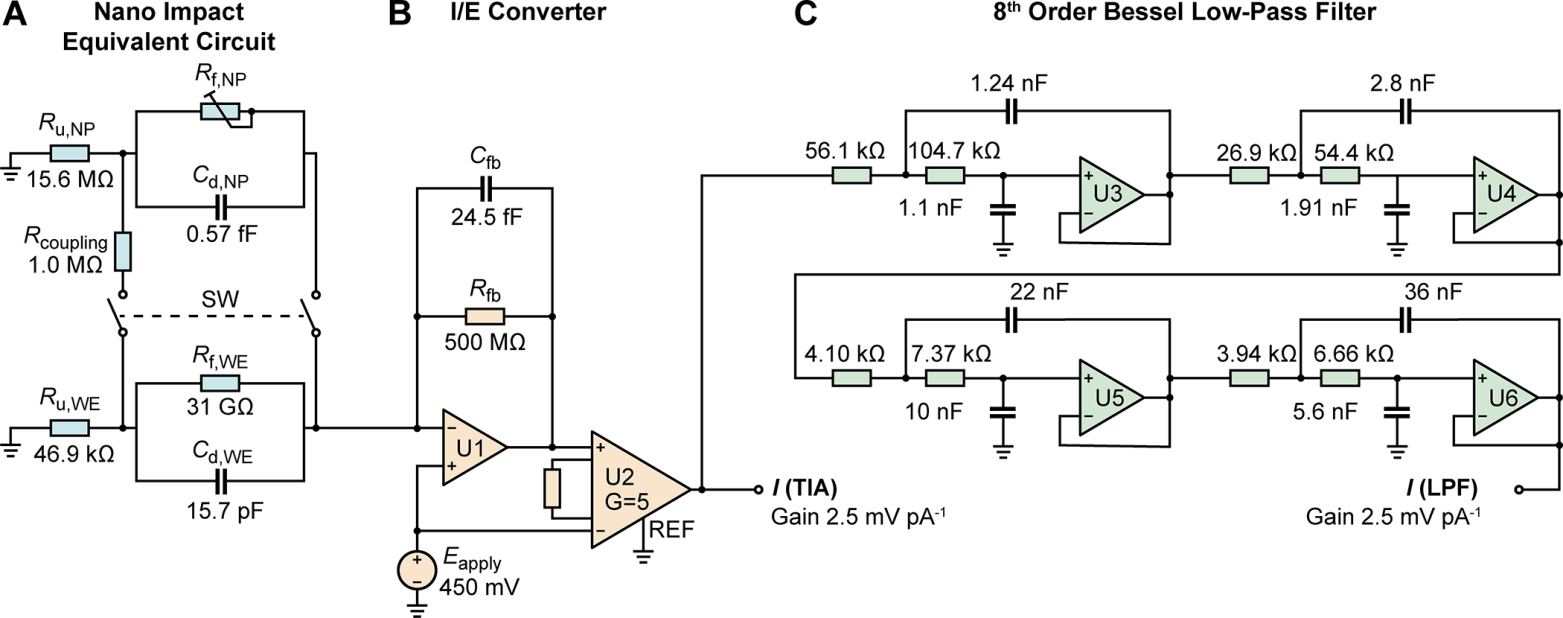
A full-system electrical circuit model for nano impact experiments consisting of the equivalent circuit of the nano impact electrochemical cell (A), the electrical model of the I/E converter (B), and the electrical model of the electronic low-pass filter (C). Each of these blocks may be customized to match the system utilized and mechanistic modeling of the nano impact experiments. The component values shown are based on the nano impact experiments of silver nanoparticles in chloride solution performed using our experimental setup with a low-pass filter cutoff frequency of 1 kHz; for further details, see the context and Supporting Information: Parameters definitions and calculations for the equivalent circuit in Figure 2.

The core of this model is the construction of an electrical equivalent circuit representation of the electrochemical cell capable of generating correct nano impact signals in accordance with the electrochemical model prediction. The equivalent circuit (Figure 2A) is composed of two parallel circuits of charge transfer resistance and double-layer capacitance, one for the working electrode and one for the nanoparticle. The switch *SW* separates these two circuits, mimicking the electron tunnelling that occurs upon nanoparticle collision. If capacitive components play an important role in the analysis, such as potentiodynamic measurements or high-capacitance systems, the double-layer capacitance may alternatively be modeled as a constant phase element. This provides more flexibility to accommodate nonideal behavior of the electrode–electrolyte interface in the simulations. Since the capacitive current contribution in our case was small compared to the faradaic current during electrooxidation, a simple capacitor was utilized to represent the double layer in the model. While the majority of the components in this circuit block can be considered static, the *R*_f,NP_ that determines the oxidative current of the nanoparticle upon impact is time-dependent and must be carefully modeled to derive the electrochemical model’s analytical solution. In the following example, we demonstrate the model’s applicability by examining the oxidation of silver nanoparticles in a chloride-ion-containing solution.
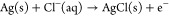
1With an excess overpotential, reaction 1 becomes mass-transport control. The steady-state diffusion-limiting current, *I*_f,NP_, then can be calculated from
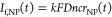
2where *k* is a prefactor that varies according to the diffusion field and electrode geometry, e.g., *k* ≈ 2.77π for a spherical nanoparticle lying on a planar electrode.^26^ As seen in eq 2, the limiting current is proportional to the nanoparticle’s radius. Since the nanoparticle dynamically decreases its size during oxidation, the limiting current also reduces proportionally to the *r*_NP_ as previously derived by Toh et al.^27^ Kätelhön et al. further derived a general solution for the specific case of the transformative nano impact experiments of a spherical nanoparticle, in which *r*_NP_ decreases with time, leading to a general solution which describes the spike shape as a function of time and the initial nanoparticle size^26^
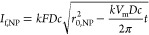
3where *r*_0,NP_ is the initial nanoparticle size and *V*_m_ is the molar volume of silver. Notably, the general solution in eq 3 is based solely on electrochemistry and does not account for instrumentation’s limited bandwidth and filtering. Using Ohm’s law, we modeled *I*_f,NP_ as a variable resistor with a resistance of *R*_f,NP_ in order to simulate the electrochemical process in electrical circuit simulations (Figure 2A, see eq 4). When the electrochemical model is connected to the signal chain comprising the I/E converter and the LPF (Figure 2B,C, respectively), the full-system simulations can predict the experimental spikes that include all the instrumentation effects. Consequently, the simulation results can be directly compared with the experimental spikes to evaluate the validity of the electrochemical diffusion-limiting model.
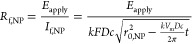
4

All electrical simulations were conducted using the transient analysis in the Electrical Simulation Program with Integrated Circuit Emphasis (SPICE) with the details provided in the Supporting Information: Electrical simulation procedures. To begin, simulations of the equivalent circuit block shown in Figure 2A (the block of nano impact electrochemical cell only, without the I/E converter and the LPF) were performed to ensure consistency between the newly developed equivalent circuit model and the analytical solution derived from the electrochemical model (eq 3). The simulated raw nano impact signals (Figure S1, see Supporting Information: Parameters definitions and calculations for the equivalent circuit in Figure 2) reveal an excellent agreement between both models. It is worthwhile to note that while the capacitive charging of the nanoparticle’s double layer can also be observed in the simulation, it does not contribute significantly to the amount of the impact charge in this experiment, and its time scale is too short to be observed experimentally due to the instrumentation’s limited bandwidth (Figure S1B).

To simulate the distortions introduced by a potentiostat, the I/E converter’s equivalent circuit is configured as a transimpedance amplifier (TIA, Figure 2B). The gain and bandwidth of the amplifier are determined by its feedback resistance, *R*_fb_, feedback capacitance, *C*_fb_, and the postamplification gain, *G*_1_.
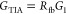
5
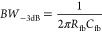
6

The values of these components have to be defined depending on the instrumental configuration. Specifically, we acquired the experimental data set using a VA-10X potentiostat (npi electronic GmbH) with gain *G*_TIA_ = 2.5 × 10^9^ V A^–1^ on which is modeled using *R*_fb_ = 500 MΩ and *G*_1_ = 5. The feedback capacitance of 24.5 fF is calculated based on the bandwidth of the instrument used in the experiment, which is 13 kHz. Following this TIA step, the output signal is routed through an eighth-order Bessel LPF (Figure 2C), which we constructed using a four-stage cascaded second-order Sallen-Key LPF.

The simulated spikes from the full-circuit in Figure 2 are shown in Figure 3 for an initial nanoparticle size *r*_0,NP_ = 15 nm and various LPF bandwidths. The simulated spikes exhibit typical elongation and delay, which are consistent with our earlier investigation utilizing the actual potentiostat system.^24^ To apply this model to each individual experimental spike, we must first determine the initial condition, which in this case is the *r*_0,NP_ of each individual nanoparticle that generates the associated impact. The nano impact approach allows us to individually access the charge used to transform each individual nanoparticle from the spike’s integral area (see eq 7 below). Using stoichiometry, Faraday law, and assuming the nanoparticle has a spherical shape, the *r*_0,NP_ can be calculated using eq 8.
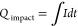
7
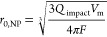
8

**Figure 3:**
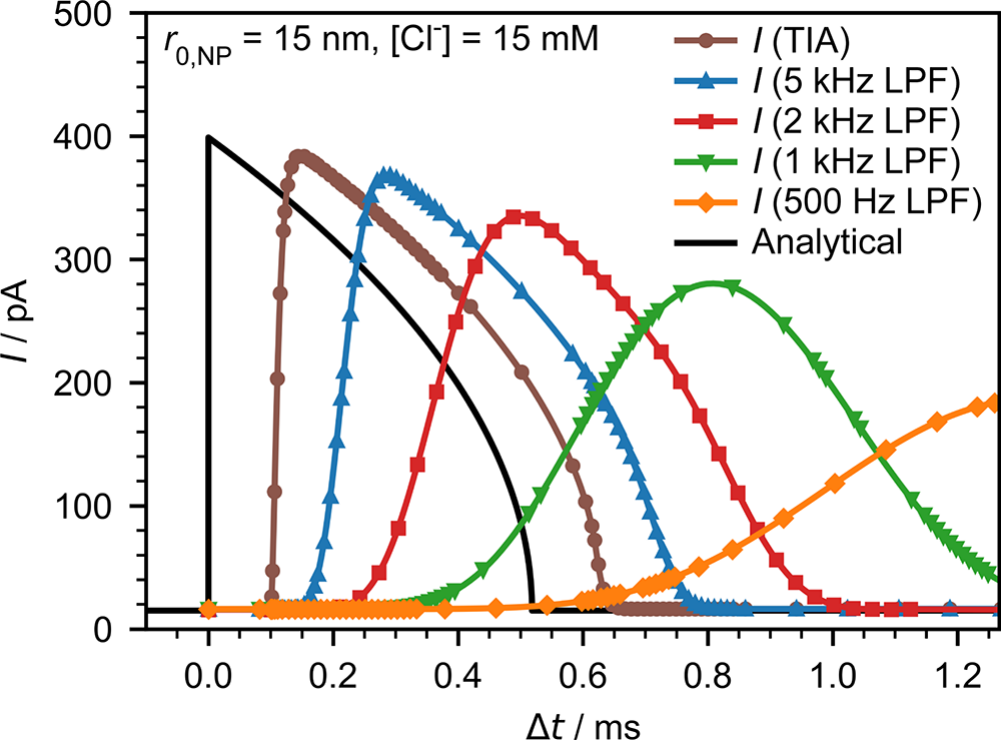
Full-system simulations of the nano impact spike using the model in Figure 2 with *r*_0,NP_ = 15 nm, [Cl^–^] = 15 mM, *k* = 2.77π, and different LPF bandwidths from 500 Hz to 5 kHz. The analytical solution using the diffusion-limited model (excluded instrumentation effects) is provided as a solid black line for comparison.

Figure 4 shows the simulations of each impact spike from the actual experiments using 29 ± 3 nm citrate-capped silver nanoparticles in an electrolyte containing 15 mM KCl and 10 mM KNO_3_ (details of the experiment are provided in the SI section S4). Notably, the simulated spikes using the shrinking sphere model (eqs 3 and 4) assuming the truncated spherical diffusion field of the nanoparticle sitting on a planar microelectrode (*k* = 2.77π) yield consistently longer spike durations compared to the experimental data obtained in this work. It indicates that the apparent rate of transformation is faster than the model prediction. Additional simulations with *k* = 4π and *k* = 2π, which correspond to hemispherical and spherical diffusion, respectively, indicate that the apparent reaction rate is more similar to the spherical diffusion case than the truncated spherical diffusion case. However, spherical diffusion is unlikely owning to the large working electrode plane truncating the nanoparticle’s diffusion field. Thus, we suggest that the apparent mass transfer, with *k* about 45% higher than expected, may be attributable to migration of chloride ions, partial oxidation or asymmetric dissolution of silver nanoparticles. Enhancement through ion migration is likely, since nano impact experiments are often performed in nonfully supported electrolyte.^28,29^ In addition to migration, electroosmotic convection might also play a role owing to the local electric field; however, it is expected to be minor, since the background current is relatively low compared to the impact current.^14,28,30^ If the oxidation of the nanoparticle is not completed during the impact, the measured *Q*_impact_ would correspond to only a fraction of the total charge required to fully oxidize the nanoparticle, leading to inaccurate calculation of the nanoparticle’s radius *r*_0,NP_. With an underestimated initial condition, the simulation will produce an impact spike with a lower peak height. This would also explain why simulations using the conventional truncated spherical diffusion model consistently yield predicted spikes with a lower peak height than the experimental data. Depending on the used electrolyte, literature reports suggest that nanoparticles may not undergo complete oxidation in a single step,^31−35^ as well as our observation that the nanoparticle size distribution determined by *Q*_impact_ is significantly smaller than the size distribution determined by transmission electron microscopy (SI, Figure S2). This study demonstrates that the application of the transient analysis using SPICE simulations allows us to consider the complexity of electrochemical nanoparticle impact experiments and to deconvolute the experimentally obtained distorted signals so as to provide valuable physicochemical insights, inaccessible from the typical, strongly simplified analytical electrochemical models based solely on the assumption of complete oxidation in diffusion-only mass-transport regimes.^26^

**Figure 4:**
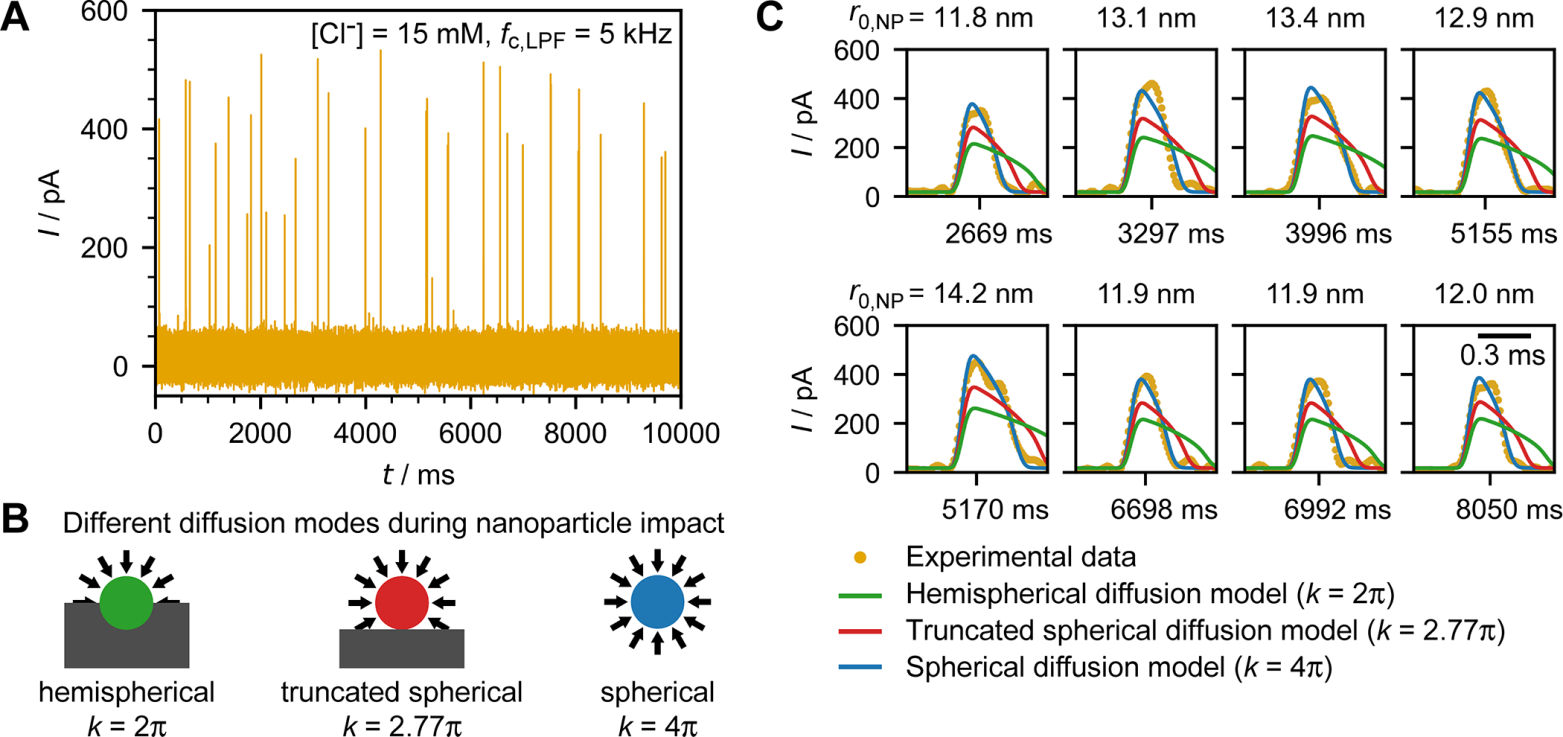
(A) Chronoamperogram of transformative nano impacts of citrate-capped silver nanoparticles in an aqueous solution containing 15 mM KCl and 10 mM KNO_3_ using a 5 kHz Bessel LPF at a sampling rate of 100 kHz. (B) Schematic illustration of the different diffusion modes employed in the simulation, whereby the truncated spherical diffusion (*k* = 2.77π) is generally accepted as a representation of the diffusion field upon nanoparticle impact. (C) Individual nano impact spikes compared to the simulation predictions using different diffusion modes revealed a faster apparent rate of transformation than predicted using the conventional truncated spherical diffusion model with *k* = 2.77π. The 0.3 ms scale bar applies to the all zoomed spikes.

In conclusion, we developed an electrical model for nano impact experiments and used it to simulate theoretical nano impact spikes which include instrumentation-induced distortions. The experimental results indicated that experimental peak durations were consistently shorter than predicted by the truncated spherical diffusion model, which is commonly used to describe this type of nanoparticle impact. This discrepancy implies that additional effects occur during nanoparticle oxidation that cannot be explained solely by diffusional flux. We proposed possible reasons for this real or apparent enhancement of mass transport during nanoparticle oxidation; migration, asymmetric or partial oxidation. With possibilities of the model modifications, the electrical simulation approach proposed in this work may open up new opportunities to gain extended insights in complex electrochemical systems by allowing for direct interpretation of convoluted experimental signal rather than missing the obscured information.

## Supporting Information Available

Summary of constants and coefficients used for calculations (Table S1), equivalent circuit of the nano impact electrochemical cell and simulation results of the nano impact spikes compared to the analytical solution (Figure S1), particle size distributions of the citrate-capped silver nanoparticles characterized by dynamic light scattering, nano impact electrochemistry, and transmission electron microscopy (Figure S2), a schematic of a unity-gain second-order Sallen-Key low-pass filter (Figure S3), coefficients for eighth-order Bessel filter design (Table S2) (PDF)
