# Supplementary material for: Electronic Circuit Simulations as a Tool to Understand Distorted Signals in Single-Entity Electrochemistry
Source: J Phys Chem Lett. 2022 Oct 21;13(43):10120–5. doi: 10.1021/acs.jpclett.2c02720 (PMC9639197; doi:10.1021/acs.jpclett.2c02720)
Supplement: Supplementary file 1 — jz2c02720_si_001.pdf [file jz2c02720_si_001.pdf]

<u>**Supporting Information**</u>

Electronic Circuit Simulations as a Tool to Understand Distorted Signals in Single-Entity Electrochemistry

Kannasoot Kanokkanchana[1], Kristina Tschulik*[1,2]

1 Chair of Analytical Chemistry II, Faculty of Chemistry and Biochemistry, ZEMOS 1.45, Ruhr University Bochum, Bochum, Universitätsstraße 150, D-44780 Bochum, Germany.

[2] Max-Planck-Institut für Eisenforschung GmbH, Max-Planck-Straße 1, Düsseldorf 40237,

Table of Contents

S1 List of symbols and summary of constants and coefficients used in this work
S2 Parameter definitions and calculations for the equivalent circuit in Figure 2
S3 Electrical simulation procedures
S4 Nanoparticle impact experiments

S1 List of symbols and summary of constants and coefficients used in this work

BW_{-3dB}	Bandwidth of the transimpedance amplifier	Hz
c	Concentration of chloride ions	mol m^{-3}
C_{fb}	Feedback capacitance	F
D	Diffusion coefficient of chloride ions	m^2 s^{-1}
E_{apply}	Applied potential at the working electrode vs. reference electrode	V
f_c	Cutoff frequency of the low-pass filter	Hz
F	Faraday's constant	C mol^{-1}
G_1	Gain of the post-TIA instrumentation amplifier	-
G_{TIA}	Gain of the transimpedance amplifier	V A^{-1}
$I_{f,NP}$	Diffusion-limiting current during nanoparticle's oxidation	C mol^{-1}
r_{NP}	Radius of a silver nanoparticle	m
$r_{0,NP}$	Initial radius of a silver nanoparticle	m
Q_{impact}	Charge transferred during the nano impact	C
R_{fb}	Feedback resistance of the transimpedance amplifier	Ω
$R_{f,NP}$	Charge transfer resistance of the nanoparticle	Ω
t	Time since starting of the impact	s
V_m	Molar volume of silver	m^3 mol^{-1}
Λ_m	Molar conductivity	m^2 Ω^{-1} mol^{-1}

Table S1 Summary of constants and coefficients used for calculations

Constant / Coefficients	Value	Unit	[Ref.]
Ag bulk density at 20°C	10.50	g cm^{-3}	[1]
	9.725×10^{4}	mol m^{-3}	converted from g cm^{-3}
Faraday constant	9.648533212×10^{4}	C mol^{-1}	NIST 2018 CODATA
Diffusion coefficient of chloride at 25°C	2.032×10^{-9}	m^{2} s^{-1}	[1]
Molar conductivity KCl at 20 mM	1.38×10^{-2}	m^{2} Ω^{-1} mol^{-1}	[1]
Molar conductivity KNO_3 at 20 mM	1.32×10^{-2}	m^{2} Ω^{-1} mol^{-1}	[1]

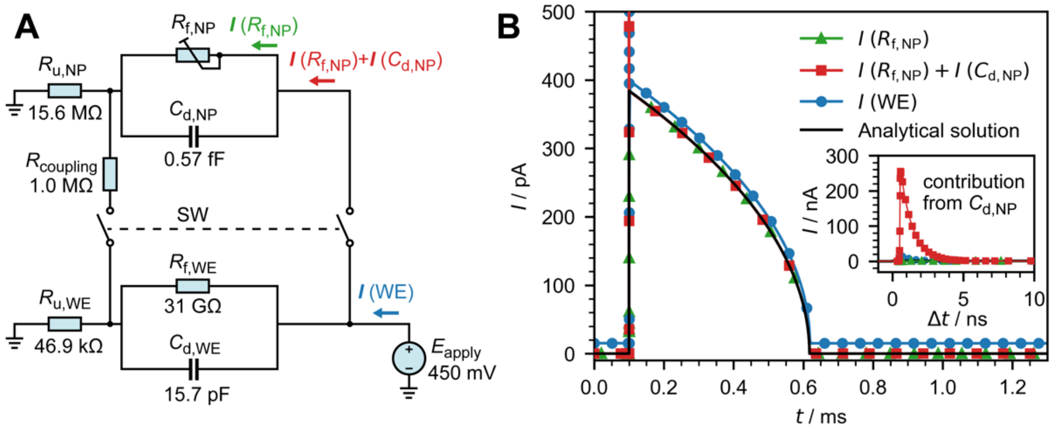

Figure S1 A) The equivalent circuit of nano impact electrochemical cell. Parameters values are calculated based on the transformative nano impact experiment of silver nanoparticles in chloride aqueous solution with r_0 = 15 nm and [Cl$^-$] = 15 mM, the $R_{f,NP}$ is defined using equation (4) and $k = 2.77\pi$. B) Simulation results of the nano impact spike compared to the analytical solution. It could be seen that the electrical simulation are in excellent agreement with the analytical solution with the difference only in the transient capacitive current from the charging of the $C_{d,NP}$ (inset) which appears only a few nanosecond after the switch *SW* is turned on and is far too short to be practically resolvable given the bandwidth of conventional low-current potentiostats.

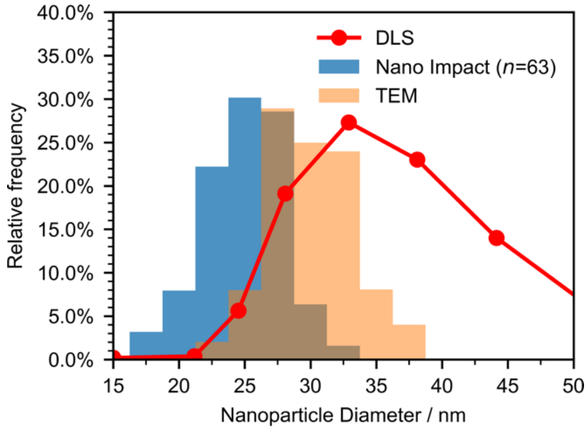

Figure S2 Particle size distributions of the citrate-capped silver nanoparticles characterized by Dynamic Light Scattering (DLS), Nano impact electrochemistry using Q_{impact} and Equation 8 (n = 63), and Transmission Electron Microscope (TEM)

S2 Parameter definitions and calculations for the equivalent circuit in Figure 2

The double layer capacitance of the working electrode, $C_{d,WE}$, and the nanoparticle, $C_{d,NP}$, are calculated using equation (S1) and (S2) given the electrode radius of 5 μm and conventionally estimated specific capacitance of 20 μF cm^{-2}. Due to the fact that the measurement is performed under a static condition and the expected $C_{d,NP}$ is extremely small (sub femtofarad), its contribution to the measured current and charge is insignificant in comparison to the faradaic current flow during nanoparticle transformation (Figure S1B).

$$C_{d,WE} = AC^0_{d,Pt} = \pi r^2_{WE} C^0_{d,Pt} \quad \text{(S1)}$$

$$C_{d,NP} = AC^0_{d,Ag} = 4\pi r^2_{NP} C^0_{d,Ag} \quad \text{(S2)}$$

The faradaic impedance of the working electrode, $R_{f,WE}$ is estimated from the background current (I_{bg}) measured during the nano impact experiment.

$$R_{f,WE} = \frac{E_{WE}}{I_{bg}} \quad \text{(S3)}$$

The $R_{u,WE}$, $R_{u,NP}$ and $R_{coupling}$ are calculated from equation (S4)[2,3] based on the molar conductivities of KCl and KNO_3 in Table S1 using 3 mm distance between the working electrode and the reference electrode, and 1 nm distance between the working electrode and the nanoparticle.[4,5] Since the molar conductivities of both salt solutions vary only slightly when their concentrations are less than 100 mM,[1] the conductivity of the mixed solution containing 15 mM KCl and 10 mM KNO_3 was estimated from the sum of their conductivities.

$$R_u = \frac{1}{4\pi \Lambda_m c r_e}\left(\frac{x}{x+r_e}\right) \quad \text{(S4)}$$

The molar conductivities of KCl and KNO_3 at 20 mM are 0.0138 m2 Ω^{-1} mol^{-1} and 0.0132 m^2 Ω^{-1} mol^{-1},[1] respectively. Therefore, the mixed solution conductivity of 0.339 Ω^{-1} m^{-1} was used to calculate the uncompensated resistance. With a 3 mm distance between the working and the reference electrode, the resistances $R_{u,WE}$ and $R_{u,NP}$ are 46.9 kΩ and 15.6 MΩ, respectively.

The eighth-order Bessel filter is constructed using four cascaded stages of a unity gain second-order Sallen-Key low-pass filter (Figure S3) with the values of R_1, R_2 of each stage calculated using equation S5 and filter coefficients provided in Table S2.[6]

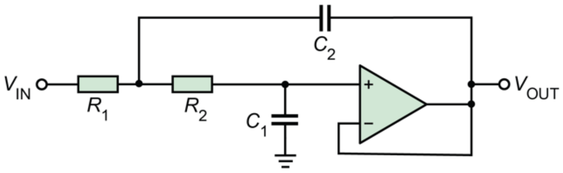

Figure S3 A unity-gain second-order Sallen-Key low-pass filter

$$R_{1,2} = \frac{a_i C_2 \mp \sqrt{a_i^2 C^2 - 4 b_i C_1 C_2}}{4\pi f_c C_1 C_2} \quad \text{(S5)}$$

Table S2 Coefficients a_i and b_i for the 8th order Bessel filter design

Filter stage (*i*)	a_i	b_i
1	1.1112	0.3162
2	0.9754	0.2979
3	0.7202	0.2621
4	0.3728	0.2087

S3 Electrical simulation procedures

Electrical simulations are performed using LTspice XVII for OS X software (Build Oct 30, 2020, Version 17.0.18.0, Analog Devices Inc.). The U1 is UniversalOpamp2 (Avol = 100 Meg, GBW = 45 Meg, Slew = 150 Meg, ilimit = 25 m, rail = 0, Vos = 0, phimargin = 45, en = 0, enk = 0, in = 0, ink = 0 and Rin = 1 T). The U2 is AD8421 (Analog Devices, Inc.). The U3 to U6 are ADA4825-1 (Analog Devices, Inc.).

All simulations shown are based on SPICE Transient Analysis (.tran) with default setting parameters (gmin = 1E-12, abstol = 1E-12, reltol = 0.001, chgtol = 1E-14, trtol = 2, volttol = 1E-6, sstol = 0.001, and mindeltagmin = 0.0001).

S4 Nanoparticle impact experiments

The transformative nano impact experiments were performed using the 29±3nm diameter citrate-capped silver nanoparticle (AgNP) suspension (0.02 mg dm^{-3},NanoXact, nanoComposix Inc.). In each measurement, 4.00×10^{-5} dm^3 of the nanoparticle suspension was mixed with 2.00×10^{-5} dm^3 of a stock electrolyte. The stock electrolyte contained 4.50×10^{-2} mol dm^{-3} KCl and 3.00×10^{-2} mol dm^{-3} KNO_3 dissolved in deionized water (Millipore, conductivity 0.055 µS cm^{-1} at 25 °C). After mixing, the concentrations of KCl and KNO_3 in the final solution were 1.50×10^{-2} mol dm^{-3} and 1.00×10^{-2} mol dm^{-3}, respectively.

All measurements were performed in a thermostated cell set to a temperature of 25.0±0.5°C using a cryostat (Alpha RA 8, Lauda GmbH). The electrochemical measurement was performed using a three-electrode setup consisting of a 10mm platinum working electrode, a leakless miniature Ag/AgCl reference electrode (ET072-1, eDAQ Pty. Ltd.), and a platinum wire counter electrode. The VA-10X analogue potentiostat (npi electronic GmbH) was used to apply a potential of 450mV vs. Ag(s)|AgCl(s)|3 M KCl(aq) and measure the current signal, which was filtered using the potentiostat's

built-in built-in Bessel low-pass filter of 8th order with various cutoff frequencies ranging from 50Hz to 20kHz. A 16-bit analog-to-digital converter (USB-1608FS-Plus, Measurement Computing Corp.) was used for data acquisition, which was controlled by the in-house developed software at a 100 ksps data acquisition rate.

References

(1) Haynes, W. M., Ed.; Lide, D. R., Series Ed.. CRC Handbook of Chemistry and Physics, 94th ed.; CRC Press, **2013**. https://doi.org/10.1201/b17118-10.

(2) Němec, L. The Effectiveness of Ir Compensation in Controlled-Potential Polarography. *J* Electroanal Chem **1964**, *8*, 166–170. https://doi.org/10.1016/0022-0728(64)87010-8.

(3) Bard, A. J.; Faulkner, L. R. Electrochemical Methods: Fundamentals and Applications, 2nd ed.; Wiley: New York, **2000**.

(4) Tschulik, K.; Cheng, W.; Batchelor-McAuley, C.; Murphy, S.; Omanović, D.; Compton, R. G. Non-Invasive Probing of Nanoparticle Electrostatics. *Chemelectrochem* **2015**, *2*, 112–118. https://doi.org/10.1002/celc.201402285.

(5) Kissling, G. P.; Bünzli, C.; Fermín, D. J. Tuning Electrochemical Rectification via Quantum Dot Assemblies. *J Am Chem Soc* **2010**, *132* (47), 16855–16861. https://doi.org/10.1021/ja106149g.

(6) Mancini, R., Ed.; Op Amps for Everyone: Design Reference, 2nd ed.; Newnes: Amsterdam, **2003**.
